# Supporter or obstructer; experiences from contact person activities among Swedish women with breast cancer

**DOI:** 10.1186/1472-6963-5-9

**Published:** 2005-01-25

**Authors:** Christina Carlsson, Mef Nilbert, Kerstin Nilsson

**Affiliations:** 1 Department of Oncology, Institute of Clinical Sciences Lund, Lund University, SE – 221 85 Lund, Sweden; 2 The Department of Research & Development, County Hospital, SE – 301 85 Halmstad, Sweden; 3 Schoool of Life Science, University of Skövde, Box 408, SE-541 28 Skövde, Sweden

## Abstract

**Background:**

Swedish patient associations for breast cancer patients (PABCPs) offer patients with breast cancer unlimited meetings with a breast cancer survivor, a contact person (CP). We applied the voluntary action perspective in this interview study with members of Swedish PABCPs in order to explore how women with breast cancer experienced their contact with a CP from a PABCP.

**Methods:**

Audio-taped narratives from 8 women were analysed using Reissman's monitoring and Gee's analysis structure.

**Results:**

Three themes appeared: 1. Shared experiences give new perspectives on having cancer, 2. Feelings of isolation are a part of the identity of the illness and 3. Relations with others enable self-help. However, the relationship with the CP is sensitive to timing, correct information and understanding.

**Conclusions:**

CPs act as sounding boards and should optimally have capacity for listening, gives support and act as partner in this conversation. On the other hand, CPs should be aware that their presence and limited general medical knowledge could at times disturb the patient's psychological recovery and strengthen feelings of isolation. Thus, PABCPs must be careful in selecting CPs and offer relevant educational activities related to the themes identified herein.

## Background

Proximity to individuals and society in order to reach out and provide optimal support is basic for patient associations for cancer patients (PACPs) [1,2]. Swedish patient associations for breast cancer patients (PABCPs) offer breast cancer patients' unlimited meetings with a breast cancer survivor, a contact person (CP). The present study focuses on women with breast cancer and their experiences from having a CP. In Sweden, an association consists of a number of individuals who work together in an organised form towards a common vision [3]. The Scandinavian concept of an 'association' is different from that in Western Europe and the U.S.[4,5]; in Scandinavia the collective rather than the individual aspect is emphasized with focus on social activities, educational workshops, and support groups [6,7]. However, also individual activities exist within the associations and the CP activities in Swedish PABCPs constitute an example hereof. This activity is inspired by the *Reach to Recovery Program *(RtoRP), a world-wide rehabilitation program initiated in the U.S. in 1952 and was later established also in Europe. RtoRP is based on the opportunity to meet with individuals with similar experiences and thereby learn different ways of dealing with disease-related feelings and problems [8,9].

Rehabilitation from breast cancer is a requirement for becoming a CP in a PABCP according to the national organisation for Swedish breast cancer associations, (BRO) [2,10]. Listening, supporting, and acting as a partner in conversations and discussions with a breast cancer patient is common to all CPs, although the forms may differ, such as one-to-one meetings or group meetings, between different PABCPs. The 28 of the total 32 PABCPs in Sweden (according to BRO) have appointed specially selected persons to coordinate the CP activities. In addition to self-rehabilitation for breast cancer, the PABCPs also provide education that includes basic psychological knowledge, medical information, organization of and contacts with the health-care system for the CPs [10]. This training program is aimed at preparing the CPs for their task to support individuals in a similar situation. Hence, the CP activity provides an opportunity for cancer patients to meet individuals with similar experiences and to learn different ways of dealing with feelings and problems [11-13].

Treatment and voluntary action provide different perspectives in studies of self-help groups or mutual support groups in the voluntary sector. We have, in accordance with other researchers' views, chosen to apply the voluntary action perspective in this study of CPs activities within the voluntary sector [14-16]. Support in self-help groups, in which the members share and articulate common experiences, is often viewed as a variant of professionally led group therapy [16-19]. However, the treatment perspective in the latter type of groups is based on the elements of the intervention that lead to cure and on the result obtained. In contrast, the voluntary action perspective of the self-help group is based on a mutual relationship with focus on benefits and aspects related to the individual or to the group [16,20,21]. What experiences do the individuals have in their contact with a CP and what do the individuals (in terms of their breast cancer illness) gain from their contact with the CPs from a PABCP? We applied the voluntary action perspective in this interview study of with the aim to explore how women with breast cancer experienced their contact with a CP from a PABCP.

## Methods

### Informants and data collection

Women with breast cancer who had personal experience from CP activities were informants in this study. To qualify for participation, the informants were required not to be involved in treatment (surgery, chemotherapy, or radiotherapy) of the primary tumour. The cancer diagnosis should be within 3 years of the data collection in order to recall the illness and treatments obtained.

We approached volunteers responsible for contact and visiting activities in 3 PABCPs who in their turn contacted 8 women who were judged eligible for the study. These women were given information on the purpose of the study and the interview procedure, and confirmation of confidential treatment of the women's information was sent by the PACP's. The recipients of the letter were informed that it was sent by the PABCP, but they were offered to contact the researcher with questions. The informants were asked to give their decision to the author (CC) by telephone within two weeks and all 8 individuals invited chose to participate in the study.

The 8 informants had a mean age of 56 (range 39–69) years and demographic characteristics are presented in Table 1. Of these individuals, 3 were diagnosed between 3 and 4 years prior to the study, but since these individuals provided extensive narratives they were allowed to stay in the study. Of the 8 individuals, 5 had experience of contact with one single CP, two of several CPs (group sessions), and one had experience of open CP meetings.

**Table 1:** Demographic characteristic of informants (*n *= 8)

**Characteristics**	**Number**
*Age:*	
39 – 69 (years)	56 (mean)
	
Married and children	8
	
*Time since diagnosis*:	
18 – 49 (month)	34 (mean)
	
*Treatment:*	
Operation	8
Cytotoxic therapy	4
Radial therapy	7
Hormonal therapy	2
	
*Experience of CP:*	
Open meetings	1
One single CP	5
Several CP	2

All the interviews were held in the home districts of the participants. The data were based on audio-taped narratives and takes into account people's natural way of constructing and interpreting experience and provides the opportunity to take relevant contextual factors into account [22]. The personal narrative refers to brief and topically specific discrete stories recapitulating specific events that the narrator had experienced [22,23] (cf Mishler [24] narratives about extra exceptional life events). The informants were asked to tell about their experiences from having a CP. The initial exhortation, "can you tell me about..." was intended to encourage the informants to tell their story in their own way, which would allow analysis from the voluntary action perspective [16]. Each narrative lasted 30 – 50 minutes, and was transcribed verbatim.

### Analysis

Transcription is a part of the analysis, regardless of whether the emphasis is on the content or the form [25]. This study focused on the contents of the stories. We used Gee's analysis structure [26], which is adapted to long oral stories, and gives attention to how a story is told and organizes the narrative into sequences, "stanzas" (poetic units) that often consist of four lines and represent a condensation of the narration. The narration also contains small interruptions that embody the basic themes. The basic theme often consists of a single line or sentence, the "coda" [26] which gives instant information about the meaning of the narration [22]. To discover the rhythm and to gain an understanding of each narration, the first author (CC) listened to the taped narratives. The two authors CC and KN thereafter read and re-read the transcribed text several times to identify the meanings (the stanzas) and the basic themes (the codas) of the stories (for examples, see Table 2). This monitoring (Riessman's term for reading and interpreting a text) emphasized how the narrative consisted of stanzas and codas [22,26].

**Table 2:** Illustrations of structured transcribed text into stanzas and codas

**Transcribed text**	**Stanza and coda**
/.../ It can have do with prostheses, cytotoxins and radiation therapy, then that I haven't had to do but then there's so much we have in common, so the pain over getting the diagnosis and experience of the operation and – worry about the future	*Stanza* There's so much we have in common pain over the diagnosis experience of the operation and worry about the future
And it feels incredibly good to be able to talk to someone who has the exact same experience, even though the health care personnel have a lot of experience – but that is – that is a different kind of experience, since it's more observational or from the outside /.../ (Informant 8)	*Coda* And it feels incredibly good to be able to talk to someone who has the exact same experience

The authors examined and discussed the stanzas and codas on the basis of their contents and relevance in relation to the aim. The final monitoring was done to find the informants' meaning according to their breast cancer when they met another individual with experience of breast cancer, i.e. a CP. The coda was used as the base in the final thematic monitoring [22] and we found 3 themes, including sub-themes, that illustrated the individuals' experience of CPs. Stanzas were used later in this text to exemplify what emerged in the themes.

Ethical permission for the study was obtained from the Ethics committee at Lund University (LU 605-01).

## Results

The thematic contents of the narratives' stanzas fell into the following themes, which were related to the purpose of the study:

1. Shared experiences give new perspectives on having cancer

2. Feelings of isolation are a part of the identity of the illness

3. Relations with others enable self-help

Themes and sub-themes are presented in Table 3.

**Table 3:** Themes and sub-themes

**Theme**	**Sub-theme**
1. Shared experiences give new perspectives on having cancer	1:1 Feelings of not being alone
	1:2 Cancer can be survived
2. Feelings of isolation are a part of the identity of the illness	2.1 Being different, unique and odd
	2:2 One among others like oneself
3. Relations with others enable self-help	3:1 Talking with others, asking questions and learning
	3:2 Right time for making contact
	3:3 The contact person provides support
	3:4 Indifference in the contact with the CP

### 1. Shared experiences give new perspectives on having cancer

#### 1:1 Feelings of not being alone

The meeting with the contact person can have emotional significance in terms of understanding that one is not alone in one's illness. Emotions came out in one case by a postcard sent by the CP. The following stanza from the informant exemplifies the CPs' activity.

I got a card from my CP

a (certain motif) where she wrote

(name) I know what you're going through

I'm thinking about you (informant 2)

The receiver kept the card and it gave her a warm feeling long after the most difficult time of the cancer illness was over.

#### 1:2 Cancer can be survived

The contribution of the cancer association and the CPs to giving the women insight about their illness can take place through invitations to rehabilitation activities, such as physical therapy groups. One woman said that, in spite of her having many questions, she did not feel ready to have contact with the association other than coming to the therapy sessions, which gave that woman sufficient and obvious evidence that she was not alone. At the water therapy sessions, she was able to see that it was possible to survive cancer and said "it's nice to be able to, like feel that fellowship". The following stanza illustrates the activity.

when we sit in the sauna – so everybody is looking -

some have no breasts

and some they've taken parts of

everybody looks a little strange (informant 7)

### 2. Feelings of isolation are a part of the identity of the illness

#### 2:1 Being different, unique, and odd

One woman said that her contact with her CP gave a long-lasting and negative experience of the meeting. After having received advice from a nurse, she had herself contacted the CP. Instead of getting access to other women's experiences through the CP she was left with an even stronger feeling of not only being ill but also of being odd. This feeling was caused by the CP not knowing that it was possible to have bilateral breast cancer at the same time (which this woman had). The CP had reacted with alarm – a reaction that frightened her and made her even more scared. These unpleasant feelings were still present after a year and because of the negative reactions she did not seek further contact with the CP, which led to a sense of embarrassment since she had initiated contact. The narrative illustrates the feelings isolation was strengthened by the CP and how the process of relating to the new disease identity was damaged.

Another narrative came from a woman who felt odd because she did not identify herself as being ill. She felt that her cancer was less serious since she did not need further treatment after the operation. This feeling of being isolated gave her a great deal to think about, which she could talk about with her CP. The reaction of being ill came late, according to the informant, and was triggered when she wanted to donate blood. When she filled in the health certificate she realized she was not permitted to do so. The CP was available at that time to talk about the incident and the following two stanzas illustrate the importance that the CP was ascribed:

it feels like she [the CP] understands

what I mean, like she understands

that I can float above it

and thought like it wasn't so serious (informant 8)

yes, I think that

she took me seriously

when I felt like it was hard

not to be able to identify myself in the group [the PACP group] (informant 8)

#### 2:2 One among others like oneself

One woman described being one among others like oneself by talking about the special feeling that came over her when she was welcomed into the association. It was not necessary to say that she had had a breast cancer operation since everyone there knew; this was obvious in the association. Another woman described the importance of meeting people in the same age and situation in life, which took place at regular theme meetings for younger women. A younger woman exemplified the importance of being the same age by saying that women of the same age had common questions to discuss – questions that could not be talked about with health professionals. The following two stanzas illustrate how the women used the CP meetings to talk with women with similar problems:

it could for example be

how I could make up my eyes now

when I don't have

any eyelashes (informant 6)

if there was anyone

who had children

because the ones I'd met were only older people

who didn't have children living at home (informant 6)

### 3. Relations with others enable self-help

#### 3:1 Talking with others, asking questions, and learning

At each contact meeting, the women felt that they could gain knowledge through the different experiences that were described about different forms of treatment and their effects on well-being. One woman describes how she could balance her worry in this environment by talking about what she thought about, which is illustrated in the stanzas below:

how will I go through this psychologically

in the future

will I trust this answer

or how much will I worry (informant 2)

alot of questions like that

that I've thought about

and that I could

talk about with other people (informant 2)

Another woman's reactions to some of the meetings were feelings of sadness, while other meetings could give her hope for the future. However, while the meetings made her react differently, she felt it was important to come to the CP meetings regularly to see with her own eyes that the other women were alive and led good lives. It came out in another narrative how it is gradually possible to talk with any of the members of the association that have experience even though it is the CP that is the person one initially talks with. Thus in the long run the experiences are felt as being most important, where the CP acts to open the door to them.

#### 3:2 Right time for making contact

Time, in the sense of a particular phase in the process, is important when the women want to make contact with others with experience of having breast cancer. One woman felt that she was not ready to contact the association and meet others with similar experience despite having many questions. At first, she said, she wanted most just to crawl into herself and manage on her own without involving other people. Nevertheless, this woman was content having received a card from the CP early in the process, telling her about the possibility of establishing a contact. The possibility of getting a contact is thus given higher priority than hearing others' experiences at an early stage.

#### 3:3 The contact person provides support

One woman evaluated the meeting with the CP positively in part because the CP was a trained CP and in part because the CP knew what she needed to hear. This is illustrated in the following stanza:

it was most of all good

to hear [name of CP]

maybe because she had training

knew what you needed to hear (informant 8)

Another woman described how she had used her CP as a "sounding board" in her choice of surgical method. It comes out in this narrative that the sounding board function and not the counselling function made the woman finally decide in favour of a surgical method that the CP had not recommended on the basis of her own experience. The fact that the CP had given an opinion helped the woman to form an opinion herself. Thus it is not necessary to share the exact same experience; at times it is enough to share the experience of cancer.

One of the women said that she would have appreciated there being someone with experience to contact when she was forced to wait for a long period before having her operation. Access to a CP for one's own personal problems is illustrated in the following stanza:

I know she's the contact person

which makes me think

that I can turn to her

and talk about my special problems (informant 8)

#### 3:4 Indifference in the contact with the CP

Women describe not only the benefit of the CP meetings but also say that there can be a feeling of more or less indifference in the contact with the CP. In one example the CP acted as a guide to the association's premises but had otherwise not spoken of anything in particular. Another woman had come into contact with the association's CP at an educational class but had never contacted a CP herself. For these women it was sufficient to contact with other women in educational classes organised by professionals.

## Discussion

Since the CP activity is central within the PABCPs, we aimed to investigate how women with breast cancer described their experiences from having contact with a CP. The narratives allowed the women to reflect and formulate themselves through this opportunity to tell their own stories with the aim to analyse their experiences [24]. The strength of the narrative method lies in studying a person's identity in times of changing circumstances as in this case, where the individual with a diagnosis of breast cancer meets another individual with the same experience [22]. The experiences described from CP meetings reflect how shared experiences give new perspectives on having cancer and how it is possible to help oneself through the relation to the CP. PABCPs offer meetings with survivors, but it may be difficult to predict the optimal time for each individual to establish such contact since individuals react differently to the diagnosis and to the cancer treatment it requires.

The narratives in our study illustrate the importance of meeting women of the same age and in a similar life situation and of being in an environment (CP meeting) that is free of need to explain. Meeting others and experiencing their' reactions help these women feel normal [16]. "Catching one's breath" from the isolation that the situation creates seems, according to the respondents, to be needed during certain periods. To become aware of not being alone with the disease, to receive visible evidence there of, and to realize that it is possible to survive appeared most important to the women in our study. The fellowship of existential uncertainty could be discerned in several of the narratives. However, although some women expressed a need to meet with others in order to reduce feelings of isolation, they did not always initiate a contact with the CP. Our results demonstrate that CPs could act as sounding boards in treatment issues and that this could be important for the woman's possibility to make her own decision. When these data have been presented during further contacts with PACPB members the findings were perceived as recognizable and the members reported similar experiences (data not shown). However, other aspects and reactions may be identified in other types of patient's associations or in other cultures. Indeed, considering the large number of PABCP in Europe multicentre studies with such a focus would be of interest [27].

Even though all patients do not wish to be confronted with the experiences of others via personal contact with a CP, they may be ready to be confronted with the disease in other ways. A confrontation with women who have undergone partial or complete mastectomies can take place in water therapy sessions and in the sauna. The results show that this meeting also gave a feeling of fellowship. This may mean that the person who chooses only this kind of activity is ready only for visual impressions. Breasts mean different things to different women, and only the individual woman can define how great a handicap breast surgery is. Langellier and Sullivan [28] studied illness narratives among breast cancer patients that showed clusters of meanings: the medicalized breast, the functional breast, the gendered breast and the sexualized breast. Compared to other research these results suggest both greater and fewer problems with femininity, sexuality and body image than have earlier been presumed.

It is also important that a CP is able to manage timing, in the sense of the phase in the process at which contact is made with others who have experience or when the person is ready to confront others' experiences. Great sensitivity towards individual needs is required and there are individual variations as to the optimal time for a meeting, which are related to when the individual is ready to encounter other people's experiences. In terms of being ready, thoughts arise as to the many visual and auditory impressions that cancer patients receive during a treatment session, e.g. in a waiting room and when given treatment, regardless of whether they are ready for these or not.

It is conceivable that some individuals do not have any need to meet with other people and share their experiences since recovery can also occur without involving others. However, it is not only the initial need for contact that varies. Women also have different needs in terms of the duration of the contact. For one person, contact with a CP may simply mean guidance in the association's activities while for another a CP can act as a support person for a period of several years and the information-seeking behaviour can also change over time [29].

For the woman who felt great isolation in the illness the meeting with the CP strengthened the feelings of isolation, which demonstrates how the meeting with the CP led to a non-intended consequence. This demonstrates the need for the PABCPs to be meticulous in the choice of CPs; the requirements must not only be that they have been rehabilitated but also that they have substantial knowledge about cancer, treatment possibilities, and psychological reactions to disease. Although self-help may come through a relation with other individuals with a shared experience [30] the associations' CPs should be aware that patients' meeting with them does not necessarily provide support, but may strengthen feelings of isolation.

The results also demonstrate that some questions could not be answered by health-care professionals, but rather by individuals with personal experience from cancer. Hence, new knowledge developed in the meeting with the CP (cf Borkmans [14] thinking concerning the meaning perspective) and it is therefore important that health-care professionals allow and perhaps also encourage cancer patients to participate in patient's associations and thereby share their experiences with others [31]. Worry was balanced by their own experience being reflected in the experience of others, which indicates that CPs have a function in areas that professionals by tradition control, such as in information about treatments and outcome.

Our findings confirm results that indicate that support in the form of social relationships with other breast cancer women empowers these women by giving them abilities to cope and adopt supportive roles towards each other during treatment [32,33]. Furthermore the emotional support that is given in connection with other survivors is important for navigating the short and long term impacts of cancer as well as the benefits from rehabilitation [34,35]. Similar observations have been made among individuals with prostate cancer, where shared experiences give reassurance, helps alleviate anxiety, and provides the participants with a more positive outlook [36] and self-help has a potential that could be strengthened in cancer care [37].

## Conclusions

Our study on patient's experience from the CP activity within PABCPs – based on narratives from 8 patients with breast cancer – show that shared experiences give new perspectives on having cancer, that feelings of isolation are part of the identity of the illness, and that relations with other individuals may enable self-help. The CP is thus important for the breast cancer patient since it helps these individuals to gain a perspective on their disease, to realize that they are not alone, to provide hope for survival, and support in the feelings of isolation that are part of the identity of cancer. From the patient's perspective, it is important that the health care system provides information on the CPs, whose responsibility it is to listen, support and act as conversational partners, and the importance of having access to other persons' experiences.

The CP serves as a counsellor and needs to have an understanding for and knowledge about the patient's needs and expectations since self-help may come about in this relation. The CPs should also be aware that their presence and a possible lack of knowledge can sometimes disturb the psychological management of the disease, and contrary to the intention, strengthen feelings of isolation. The CP must also be ready to confront others' experiences, but also needs to understand that not all individuals have such a need. The CP must be able to offer many different kinds of help related to feelings of isolation, survival, and rehabilitation, and may thereby act as a sounding board for women's experiences and a shelter for emotional expressions. Hence, to optimise rehabilitation for individuals with breast cancer, PABCP should carefully select and educate CP's, and an exchange of experiences between the PABCP's and the health care system may contribute to this process.

## List of abbreviations

PABCP Patient associations for breast cancer patients

CP Contact person

## Competing interests

The author(s) declare that they have no competing interests.

## Authors' contributions

CC: study design, data collection, data analysis, and writing the manuscript.

MN: supervising the study and participation in writing the manuscript.

KN: study design, data analysis, supervising the study, and participation in writing the manuscript.

## Pre-publication history

The pre-publication history for this paper can be accessed here:


